# Endoscope ear pick: An emerging but neglected medical device

**DOI:** 10.3389/fmed.2022.977554

**Published:** 2022-11-15

**Authors:** Wendi Huang, Ying Li, Juan Huang, Yong Luo, Nanqu Huang

**Affiliations:** ^1^National Drug Clinical Trial Institution, Third Affiliated Hospital of Zunyi Medical University, The First People's Hospital of Zunyi, Zunyi, China; ^2^Department of Pediatrics, Third Affiliated Hospital of Zunyi Medical University, The First People's Hospital of Zunyi, Zunyi, China; ^3^Department of Pharmacology and Chemical Biology, Shanghai Jiao Tong University School of Medicine, Shanghai, China

**Keywords:** endoscope ear pick, otoscope, cerumen, smartphone-enabled otoscope, conventional otoscope

## Abstract

Earwax (cerumen), a normal bodily secretion, can become a problem when it obstructs the ear canal. Earwax removal is a difficult task for specialists because of the ear's unique location and the ear canal's intricate structure. Using ear scoops or cotton swabs to dig out ear wax in daily life is like “a blind man walking on a cliff.” Improper operation may damage the ear canal or the eardrum. Thus, we need a pair of visible “eyes,” otoscopes, to help us see earwax intuitively. As opposed to traditional otoscopes, which only serve as a visual aid, the endoscopic ear pick allows us to not only view the ear canal but also remove wax or other obstructions from the ear. In this review, we discussed endoscope ear pick pros and cons and discussed their future role.

## Introduction

Earwax, made from the cerumen glands in the external auditory canal, is a hydrophobic, waxy substance that provides mechanical and microbial protection to the skin of the external auditory canal and adheres to foreign objects (1, 2). According to the different components of the secretion, cerumen is classified into two types: dry and wet (3, 4). These two types of cerumen differ in color and consistency: the flocky and gray dry cerumen and the sticky yellow to a brown wet one. Under normal circumstances, earwax can be excreted by chewing and other movements aided by jaw movement without deliberate cleaning. This makes cerumen seem harmless. However, diminished self-cleaning habits and/or overactive secretion of the cerumen glands can lead to the accumulation of cerumen in the ear canal, resulting in cerumen impaction that may cause tinnitus (5, 6), aural fullness, vertigo, itching, ear pain, external otitis, and hearing loss (1).

**Figure 1:**
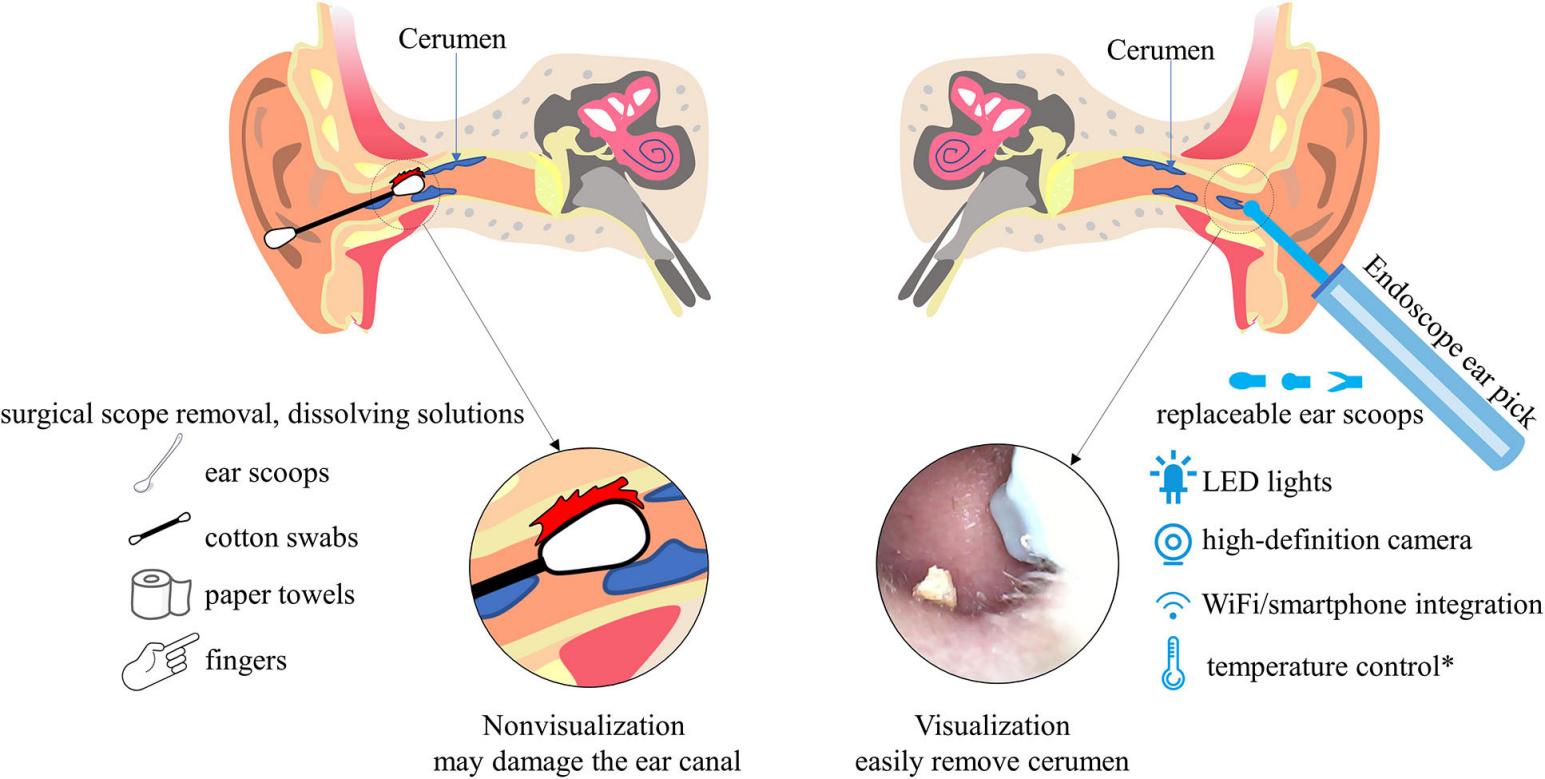
The location and general concept behind cerumen removal: From the traditional way to the Endoscope ear pick. *Most Endoscope ear picks are equipped with replaceable ear scoops, LED lights, high-definition cameras, and WiFi/smartphone integration. Only a few can control the temperature and maintain a constant temperature.

Earwax removal, whether performed by yourself or a professional, can be a challenging experience due to the confined space and low light of the ear canal. It is like playing hide-and-seek with earwax in the complex curved ear canal; cleaning earwax is primarily a matter of feeling and luck. However, we cannot observe the specific situation in the ear canal, and it is hard to see what is going on in the ear, even when looking for other's help. People commonly use ear scoops, cotton swabs, paper towels, or even fingers and various small objects that can be easily found, pushing cerumen deep into the ear canal and even damaging the ear canal (Figure 1). Moreover, individuals with intellectual disabilities and the elderly tend to have a high incidence of excessive/impacted cerumen (1). Therefore, surgical scope removal, suction, and lavage/wash are excellent ways for a doctor to operate when dealing with severe cases of cerumen impaction (5). Less severe cerumen can usually be removed at home with cerumen-dissolving solutions, oil, hot water (softened hardened wax), cotton swab, etc (7). However, compared to other treatments, manual removal necessitates tools that are not usually available in primary care clinics and greater technical knowledge (8).

Despite all these advantages, the examination of the ears was not considered an essential part of physical examination before the early ninetieth century. The otoscope has evolved from a simple cone-shaped structure without a light source to the contemporary one-electronic otoscope since 1821 when French doctor Gaspard Itard first began drawing attention to ear ailments. In addition, during the COVID-19 epidemic, many patients with ear diseases required long-term follow-up and observation after surgery or treatment, making it challenging to complete return visits on time. Thus, an excellent way to clean the earwax and let doctors help with remote diagnosis and treatment is also needed (9). With the gradual development of electronic otoscope technology, the smartphone otoscope may become an emerging technology to promote the development of otolaryngology and pediatrics. Therefore, noticing cerumen impaction and removing earwax has become a necessity.

## Structure of the endoscope ear pick

The endoscope ear pick is mainly composed of a replaceable ear pick, a micro camera, LED lights, and a grip (battery included). Some endoscope ear picks use a wide-angle professional-grade lens for wide coverage and earwax removal. Some are also equipped with a high-definition camera, and the full HD resolution can provide super clear images. Some of the ear picks are made of smooth silicone material, which is neither too hard nor too soft. When used at the right angle, earwax can be removed without damaging the ear canal's delicate skin. However, replacing the replaceable ear pick regularly is still recommended to reduce the risk of infection.

Furthermore, one does not have to worry about getting too hot or too cold. Some endoscope ear picks have temperature control. The built-in system controls it at a constant temperature. If the endoscope ear pick has WiFi, an integrated smartphone device, a user can download a particular program, connect their smartphone to the WiFi of the otoscope, and begin using it with a single click. It works with Android and iOS devices. You can also use it on a tablet. It is convenient for daily inspection of the health of the ears, nose, mouth, throat, etc. The user can also care for the health of their family, friends, or pets with this device. Its mini-size design makes it small and portable, suitable for travel and outdoors. The endoscopic ear pick is readily available on Amazon and Taobao, and its price is roughly equivalent to that of multiple otoscopies.

## Benefits of the endoscope ear pick

In recent years, endoscope ear picks have been widely produced and used to meet this demand (10). Unlike the more expensive electronic otoscopes used in hospitals, the most common pen-shaped endoscope ear pick, which wirelessly transmits image information to mobile phones and portable devices, is low-cost and easier to carry. Yet, because of its affordability and portability, it is easy to overlook that this emerging electronic device is a medical device. The picture taken of the ear canal is transmitted to the mobile phone screen in real time. This way, people can remove earwax while watching it on their phone screen. At the same time, its scoop can be disassembled and replaced, not only allowing it to adapt to more application scenarios but also facilitating disinfection and cleaning. In addition, the endoscope can function as a mini otoscope. When we encounter problems such as sudden hearing loss, we can first use the endoscope to see what is inside the ear.

In addition, this device could have unexpected uses, such as examining people's mouths, noses, and other dark, cramped places. Several studies have affirmed the value of portable smart otoscope devices as effective diagnostic and telemedicine aids in clinical settings. Freitag et al. (11) successfully used a smartphone otoscope as an alternative to tracheal intubation of rabbits in more than 30 rabbits without any complications. Experienced anesthesiologists face a challenging problem while administering anesthesia to rabbits because of their larger incisor teeth, narrower mouth opening angle, and smaller tracheal diameter compared to other species. However, this small smart device has no complications like tracheal or esophageal laceration, laryngospasm, or failure to view the larynx (11). Hakimi et al. (12) conducted research between conventional otoscope (CO) and smartphone-enabled otoscope (SEO) in first- and second-year (pre-clinical) medical students and held a view that the SEO, improving medical students' self-confidence, is a useful teaching aid for pre-clinical otoscopy training. Garrett Ni believes that a smartphone-adaptable otoscope can help pediatric residents identify ear pathology (13).

In addition, smartphone-enabled wireless otoscope-assisted online telemedicine during the COVID-19 outbreak (14). Images captured by the device could also be incorporated into electronic medical records and transmitted to facilitate remote diagnosis and management (15). Therefore, an endoscope ear pick is a potential portable medical detection and treatment device based on the ability to visualize and clean narrow cavities.

## Some possible problems

Like every coin has two sides, endoscope ear picks also have downsides. Endoscopic ear picks are generally equipped with a variety of replaceable ear scoops. This interchangeability leads to a problem with endoscope earpicks that is rare with conventional ear scoops: replaceable scoops fall inside the ear canal. As with endoscopes, skilled use requires training; otherwise, it can be harmful. Similar to performing your own endoscopy, it is not as simple as it may appear. However, its operation is still very convenient. It does not require a medical degree or skill test, but one should read the operation manual carefully before using it, mainly to be familiar with the coordination and synchronization of the screen and operation.

In addition, visualization amplifies the need for earwax cleaning, allowing people to clean the ear canal more frequently in a short period. Especially without careful disinfection of the pick, there is a risk of damage to the ear canal by inserting a non-sterile pick (16). An appropriate amount of cerumen has important physiological functions, such as covering and protecting the delicate ear canal skin and killing some microorganisms (17). Under normal circumstances, earwax does not need to be frequently cleaned. This deliberately created demand can lead to some undesirable results. Too little earwax can also be harmful. It increases the chance of infection and may cause the canal to itch (18). In addition, smartphones and Internet use remain restricted in some remote rural areas and among older people. Moreover, while doctors provide the convenience of telemedicine services through the otoscope APP, there are difficulties in identifying the patient's identity and problems related to medical insurance reimbursement (15).

## Conclusion

Overall, the endoscope ear pick is a portable medical device with more advantages than disadvantages. This device can be used not only as a mini portable endoscope to observe the internal conditions of the ear canal, oral cavity, etc., but also to clean earwax. However, it is important to pay attention to the following when using an endoscope ear pick: 1. Be sure to tighten the replaceable scoops; 2. Disinfect the replaceable scoops; 3. Before the operation, perform simulation exercises; 4. Do not use it frequently and excessively to clean the ear canal. This new type of device has been neglected. If it can be used to remove the nasal foreign body, is it possible to add some functionality to make it more usable, such as adding the styles of replaceable ear scoops, including cones, tweezers, and grippers, making it easier to remove the earwax or foreign matters, or add the function of detecting temperature to measure ear temperature?

We hope for additional studies on the rising popularity of this medical device.

## Author contributions

WH and YLi contributed equally to this work and wrote the manuscript. WH, YLi, JH, YLuo, and NH contributed to the critical revision of the manuscript and read and approved the submitted version. All authors contributed to the article and approved the submitted version.

## Funding

This work was supported by the funds of the Zunyi Science and Technology Bureau (Nos. 2017-29, 2020-107, and 2021-274) and the Science and Technology Fund of the Guizhou Provincial Health Commission (No. gzwjkj2019-1-064).

## Conflict of interest

The authors declare that the research was conducted in the absence of any commercial or financial relationships that could be construed as a potential conflict of interest.

## Publisher's note

All claims expressed in this article are solely those of the authors and do not necessarily represent those of their affiliated organizations, or those of the publisher, the editors and the reviewers. Any product that may be evaluated in this article, or claim that may be made by its manufacturer, is not guaranteed or endorsed by the publisher.
